# Inflammation-Related Epigenetic Modification: The Bridge Between Immune and Metabolism in Type 2 Diabetes

**DOI:** 10.3389/fimmu.2022.883410

**Published:** 2022-05-06

**Authors:** Qiyou Ding, Zezheng Gao, Keyu Chen, Qiqi Zhang, Shiwan Hu, Linhua Zhao

**Affiliations:** ^1^ Department of Endocrinology, Guang’ anmen Hospital, China Academy of Chinese Medical Sciences, Beijing, China; ^2^ Institute of Metabolic Diseases, Guang’ anmen Hospital, China Academy of Chinese Medical Sciences, Beijing, China; ^3^ Graduate School, Beijing University of Chinese Medicine, Beijing, China

**Keywords:** type 2 diabetes mellitus (T2DM), inflammation, macrophages, DNA methylation, histone modifications, non-coding RNA

## Abstract

T2DM, as a typical metabolic inflammatory disease, is under the joint regulation of environmental factors and genetics, combining with a variety of epigenetic changes. Apart from epigenetic changes of islet β cells and glycometabolic tissues or organs, the inflammation-related epigenetics is also the core pathomechanism leading to β-cell dysfunction and insulin resistance. In this review, we focus on the epigenetic modification of immune cells’ proliferation, recruitment, differentiation and function, providing an overview of the key genes which regulated by DNA methylation, histone modifications, and non-coding RNA in the respect of T2DM. Meanwhile, we further summarize the present situation of T2DM epigenetic research and elucidate its prospect in T2DM clinical diagnosis and treatment.

## Introduction

The global prevalence of type 2 diabetes mellitus (T2DM) is increasing rapidly (1–3). Yet, there is no marketed therapeutic drug that indefinitely cures or prevents the occurrence and progression of diabetes (4). Glucose-lowering agents alone have limited efficacy in preventing T2DM progression (5). Thus, with rapidly increasing rate of diabetes, a deeper understanding of its underlying molecular mechanisms is urgently needed to develop better therapies.

Several lines of evidence suggest that chronic activation of pro-inflammatory pathways in insulin target tissues, such as the adipose tissue, liver, muscle, and pancreatic islets may contribute to obesity, insulin resistance (IR), and T2DM (6). These evidence contributed to the term immunometabolism, which highlights the potential interplay between immune functions and metabolic defects (7). Meanwhile, persistent chronic inflammation can lead to scarring, decreased function, and organ failure, eventually leading to a rise in diabetes-related deaths (8). The vital role of inflammation in the occurrence and progression of diabetes has sparked great interest in exploring immune imbalances as therapeutic targets (4, 9).

The first trigger(s) of immune imbalance accompanying metabolic dysfunction have not been revealed, but the involvement of epigenetic modifications in the upstream regulation of inflammation has been recognized (10). Environmental factors such as altered nutritional status can induce epigenetic changes (11). Subsequently, pre-existing epigenetic marks in genes activate or repress gene transcription in response to environmental stimuli. Thus, epigenetic changes serve as key bridges in the complicated interaction between the environment and genetics to actuate the inflammatory reaction accompanying metabolic disorders.

In this review, we explain the relationship between epigenetics, inflammation and T2DM, and discuss the potential mechanisms by which epigenetic factors contribute to diabetes by regulating inflammation remodeling. We also highlight how the rapidly increasing knowledge base of epigenetics can open a door of opportunities to improve the clinical management of diabetes.

## Chronic Inflammation Caused by Immune Imbalance in the Pathogenesis of T2DM

In the past, metabolism and immunity were often considered as two separate phenomena, wherein the main function of metabolism was to maintain the transformation of the body’s substances and energy while that of immunity was to protect against foreign invaders and remove hazardous substances produced by the body itself. Recently, with a more comprehensive understanding of metabolism and immunity, the cross-talk and interaction between the two in the body’s physiological state and during disease development have been recognized (12, 13).

First, immune system function is based on the proliferation and differentiation of immune cells as well as on various types of cytokines. Immune cells need metabolic support to provide energy and substrates, such as glucose, amino acids, phospholipids and fatty acids; thus, the immune system controls metabolism to ensure its energy supply (14, 15). Cytokines in immunometabolism not only play a role in immune regulation but also act as agents of energy metabolism. Some metabolites, such as glucose, can also activate a response to pathogens as a signal medium, besides supplying energy. Overall, immunity plays a vital role in metabolic homeostasis, and an imbalance in immunity could lead to metabolic diseases including T2DM.

T2DM occurrence is consistent with an alternative immune cell profile, including changes in the mononuclear macrophage (Mϕ) system, B lymphocytes, T lymphocytes, natural killer (NK) cells, and innate lymphoid cells (ILCs). Overall, the alteration of the immune cell profile showed a pro-inflammatory proliferation, differentiation and phenotypes, including the increase in M1-like Mϕs, T helper (Th)1 cells, Th17 cells, CD8+ cells, antibody-producing B-2 cells (16–18), and downregulation of M2-like Mϕs, Th2 cells, regulatory T cells (Treg), IgM-producing B-1 cells, and ILCs subsets (such as ILC2s and ILC3s) (19–21). This immunocytic cross-talk causes pathogenic inflammation *via* the release of cocktail pro-inflammatory cytokines (such as tumor necrosis factor (TNF)-α, TNF-β, interleukin (IL)-1β, IL-2, IL-6, IL-17, interferon (IFN)-α, IFN-γ) and IgG in circulation and glycometabolic tissues such as adipose tissue, liver, muscle, and pancreas, which further disturbs metabolism, ultimately resulting in pancreatic β-cell dysfunction, glucose intolerance, and IR (4).

Mϕs, an important component of innate immunity, plays a crucial role in metabolic inflammation in T2DM. In humans, Mϕs can usually be divided into tissue-resident Mϕs, such as resident intestinal Mϕs, adipose tissue macrophages (ATMs) and liver macrophages (Kupffer cells, KCs), and recruited Mϕs, which are derived from blood monocytes through the binding of monocyte chemoattractant protein-1 (MCP-1,also named CCL-2) and C-C chemokine receptor 2 (CCR2) (22). Primarily, gut microbial composition and metabolic function could be disturbed by a high-fat diet (HFD), and the subsequent upregulation of lipopolysaccharide (LPS) could directly stimulate resident intestinal Mϕs to transfer into M1-like Mϕs, and promote the secretion of pro-inflammatory cytokines such as IL-1β, IL-6, TNF-α, and chemokines CCL-2. Meanwhile, intestinal epithelial cells can also produce CCL2 and cooperatively recruit blood monocytes into gut lumen. The monocytes gradually differentiate towards into resident mature M1-like Mϕs with loss of Ly6C/CCR2 and gain of CD64/MHII expression, and also show high levels of IL-1β, IL-6, or TNF-α and hyper-responsiveness to inflammatory stimuli (23). Through a similar mechanism, because of increased fatty acids, a hypoxic environment and local stimulation of TNF-α, IL-1β, IL-6, and other pro-inflammatory cytokines, resident tissue Mϕs transform to M1-like Mϕs in the adipose, liver, muscle tissue, and pancreatic islets. Furthermore, it contributes to blood monocyte recruitment into these tissues and converts them into M1-like Mϕs to promote an inflammatory environment. It inhibits the insulin pathway, causes IR, and impairs insulin production (24, 25). In the intestine, a HFD induces a pro-inflammatory shift in T cells, characterized by an increase in INF-γ^+^ Th1 and CD8^+^T cells, and a subsequent decrease in IL-10^+^ Tregs and IL17^+^ Th17 cells (26). In addition, there is a decrease in IgA^+^ antibody-secreting B cells and ILC3s in HFD-fed mice, and the reduced levels of colonic secretory IgA+ and IL-22 derived from ILC3s may be associated with IR (26, 27). In adipose tissue, CD8+ T cells are more abundant and can promote monocyte recruitment and differentiation into M1-like ATMs (16). Pro-inflammatory Th1 cells are also increased in obese adipose tissue leading to pro-inflammatory cell infiltration by the production of IFN-γ (28). ILC2s and eosinophils regulate adipose immune homeostasis by inhibiting M1 polarization of Mϕs based on the secretion of IL-13 (derived from ILC2s) and IL-4 (derived from eosinophils) and alleviate adipose inflammation (29). The aggregation of free fatty acids released by the liver and adipose tissue can simultaneously lead to an increase in neutrophils and CD8+ T cells, thereby inducing liver inflammation (30). Neutrophil elastase can be released and taken up by hepatocytes (31), promoting the intracellular degradation of insulin receptor substrate 2 (IRS-2), thereby enhancing IR in hepatocytes.Overall, in T2DM, the cross-talk between metabolism and immunity has been recognized by researchers, and the immune mechanism of T2DM has been partially revealed. However, in the context of T2DM, the internal homeostasis of immunity and its interaction mechanism with glycolipid metabolism need to be further explored.

## Epigenetic Changes: New Insights Into the Development of T2DM and Diabetes-Related Inflammatory Status

The Diabetes Control and Complications Trial (DCCT) (32) showed that intensive glycemic control could better delay theprogression of microvascular complications, compared to conventional therapy in patients with T1DM. During the following observationalEpidemiology of Diabetes Intervention and Complications (EDIC) study (33), patients who had been in the conventional therapy group were also switched to receive intensive glycemic control, and both groups successfully achieved similar mean hemoglobin A1c (HbA1c) levels of approximately 8%. Nevertheless, the risk of macrovascular and microvascular complications in patients with early intensive glycemic control in DCCT was significantly higher than that in patients without early intensive treatment (34–37). This has been explained by a phenomenon called “metabolic memory”, which suggests that a ‘memory’ of previous glucose exposures in target cells causes its deleterious effects to persist long after glycaemic control has been established. Growing evidence suggests that epigenetic alterations in target cells are an important cause of high-glucose ‘memory’. Thus, epigenetics, as a link between metabolic memory and the occurrence and development of diabetes, has attracted considerable attention.

**Figure 1:**
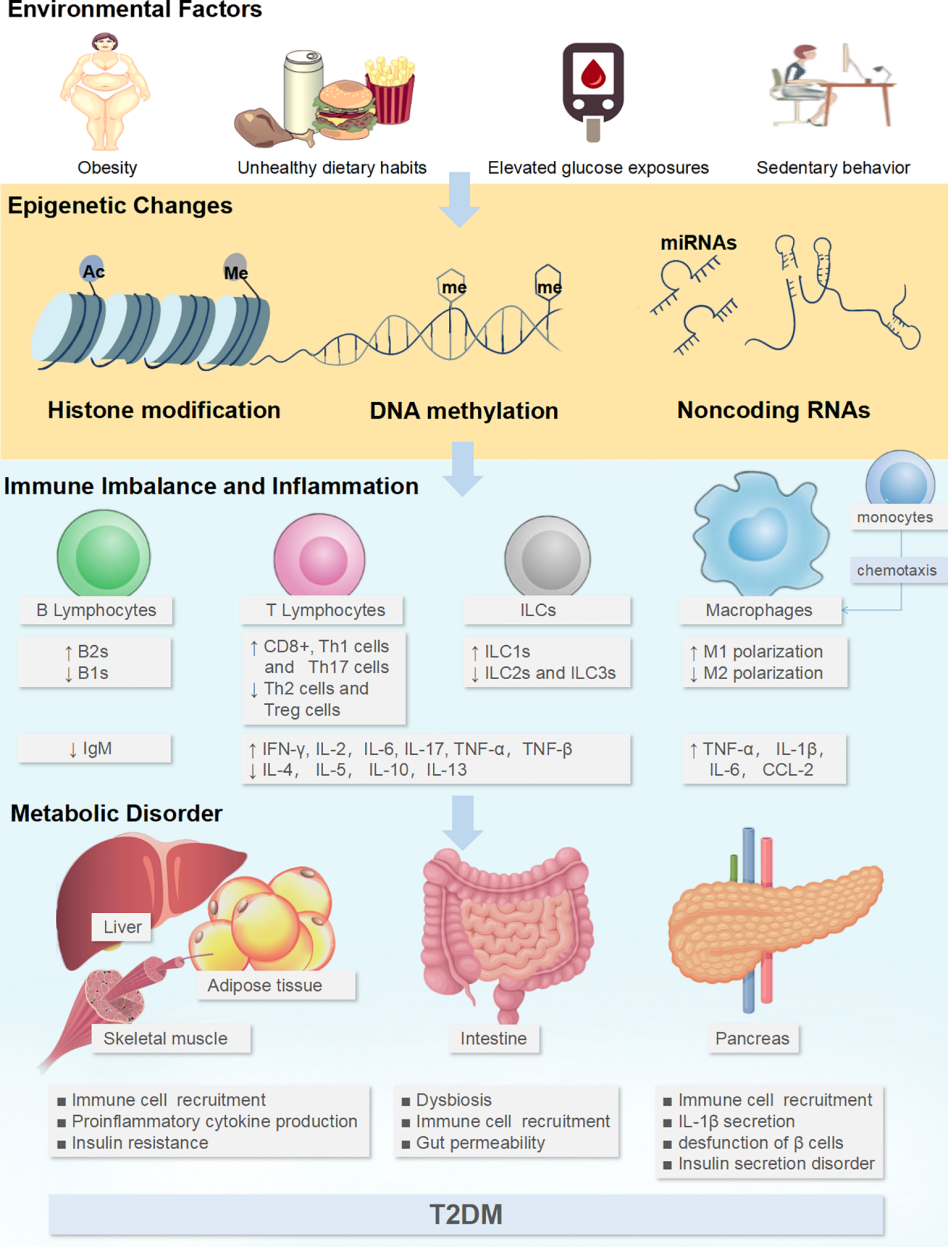
Environmental factors could affect the epigenetics of immune cells to generate tissue inflammatory states and induce metabolic disorders. Adverse environmental factors could induce or aggravate the pro-inflammatory immune cell profile through the epigenetics, including the increase in M1-like Mϕs, Th1 cells, Th17 cells, CD8+ cells, ILC1s, B-2 cells, and the downregulation of M2-like Mϕs, Th2 cells, Treg, ILC2s, ILC3s and B-1 cells. This immunocytic cross-talk causes pathogenic inflammation *via* the release of cocktail pro-inflammatory cytokines (TNF-α, TNF-β, interleukin (IL)-1β, IL-2, IL-6, IL-17, IFN-α, IFN-γ) in intestine and glycol-metabolic tissues such as adipose tissue, liver, muscle, and pancreas, which further disturbs metabolism, ultimately resulting in pancreatic β-cell dysfunction and IR. ↑: up-regulated expression, ↓: down-regulated expression.

Epigenetics refers to the heritable modifications in gene expression without changes in the DNA sequence that regulates cell differentiation, cell-specific gene expression, parental imprinting, X chromosome inactivation, and genomic stability and structure. Epigenome encompasses genome-wide DNA methylation, histone modifications, and the expression of small non-coding RNAs, primarily microRNAs (miRNAs), and also chromatin accessibility (10). As a mechanisms linking environmental factors to altered gene activity, epigenetic processes could be dynamically altered under the influence of short-term and/or long-term environmental exposures, such as drugs, diet, sedentary lifestyle, obesity, elevated blood glucose levels, and aging. Researchers have found hundreds of epigenetic alterations in relation to inflammation, obesity, and T2DM in human tissues that are relevant to metabolism. Of these, epigenetic alterations directly involved in IR and impaired insulin secretion have been reviewed elsewhere (38–40) and will not be covered in the present review. In this review, we focus on epigenetic alterations associated with immune inflammation in the context of T2DM (**Figure 1**).

As the most extensively studied epigenetic mechanism, DNA methylation regulates gene expression and maintains chromosomal stability. DNA methylation mainly refers to the covalent bonding of a methyl group, namely, 5-methylcytosine (5mC) at the CG context or the so-called CpG sites on cytosine, under the control of specific enzymes known as DNA methyltransferases (DNMTs) including DNMT1, DNMT3A, and DNMT3B (41). Methylation of DNA at gene promoter regions is typically associated with gene suppression through different mechanisms, including the recruitment of transcriptional repressors and interference with transcription factor binding. DNA methylation can be reversed by ten-eleven translocation proteins that convert 5mC to 5-hydroxymethylcytosine. A growing body of research suggests a link between DNA methylation and diabetes (42). Identified by epigenome-wide association studies, differentially methylated CpG sites annotated to several candidate genes for T2D, such as *ABCG1*, *SREBF1*, *TXNIP*, and *LGALS3BP*, have been found in the bloodc (43). Increased methylation in the *ABCG1* and *SREBF1* genes, and the subsequent downregulation of their expression, are thought to be associated with T2DM. Both ABCG1 and SREBF1 are transcriptional activators that are required for lipid homeostasis. ABCG1 mediates cholesterol efflux to mature HDL (44). Studies have shown that ABCG1 deficiency in myeloid cells promotes activation of the NLRP3-inflammasome and atherogenesis (45). In addition, decreased methylation of the TXNIP gene is also robustly associated with prevalent T2DM. TXNIP is a key regulator of oxidative stress and has been linked to inflammation; its upregulation can also facilitate the activation of the NLRP3-inflammasome and the release of inflammatory mediators (46). LGALS3BP encodes a secretory glycoprotein regulated by the NF-κB pathway. Previous studies have observed an upregulation of *LGALS3BP* in TNFα-treated adipocytes, promoting macrophage recruitment, suggesting a potential pro-inflammatory action of LGALS3BP (47, 48). In addition, in a study that focused on the methylation of genes involved in inflammation in the peripheral blood of obese and lean individuals, 28 significantly hypomethylated proinflammatory genes were found in obese individuals. Fifteen of these genes, including *CXCL6*, *TLR5*, *IL6ST*, *EGR1*, *IL15RA*, and histone deacetylase (*HDAC*) 4, showed significantly high mRNA levels. The degree of methylation was negatively correlated with fasting plasma insulin, serum IL6, C-reactive protein, and arteriolar reactive oxygen species (49). Since epigenetic patterns are cell-specific, other insulin target tissues such as adipose tissues (AT) are also of concern. Combining the transcriptomes and methylomes of subcutaneous adipose tissue (SAT) and visceral adipose tissue (VAT) samples from obese T2DM individuals and using tissue-specific regulatory networks, Jing et al. identified epigenetically dysregulated gene modules in adipose tissues. Two SAT modules were predicted to be involved in regulating adipocyte differentiation and promoting obesity-related inflammatory responses to impair insulin signaling (50).

Histones are highly conserved proteins in eukaryotic cells and nucleosomes and DNA are the basic units of chromatin structure. Histone modifications, including acetylation, methylation, and phosphorylation, which occur on the N-terminal tails of histones H3 and H4, are important for gene regulation. It can regulate gene expression by altering the affinity of histones to DNA, thereby enabling the transformation of chromatin between loose and dense states. Histone methylation and acetylation have been found to play important roles in the regulation of several key genes associated with diabetes. Histone acetylation states is catalyzed by histone acetyltransferases (HATs) and HDACs. Generally, histone lysine acetylation is associated with transcriptional activation, whereas acetylation removal is associated with transcriptional repression (51). The sirtuin (SIRT) family of deacetylases, specifically SIRT1, regulates several factors involved in metabolism, including adiponectin secretion, inflammatory responses, and levels of reactive oxygen species, which together contribute to the development of IR (52). Histone methylation, in contrast to acetylation, is more constant and long-standing and is associated with both active and silent genes depending on the specific location and degree of modification. Despite being relatively stable, histone methylation can be dynamically modified through the concerted actions of histone methyltransferases and histone demethylases (53). Together with the increased histone methyltransferase Set7 expression in peripheral blood mononuclear cells, the Set7-dependent monomethylation of lysine 4 of histone 3 on NF-kB p65 promoter was also found in patients with T2DM. This epigenetic changes were related to vascular dysfunction *via* upregulation of NF-kB subsequent transcription of oxidant-inflammatory genes, and increased plasma levels of intercellular cell adhesion molecule-1 and monocyte chemoattractant protein-1 (54). Animal experiments revealed that a series of genes, mainly those enriched in the MAPK signaling pathway, in adipose tissue from HFD-fed mice were activated by histone 3 lysine 9 methylation (H3K9me)2, H3K9me3, H3K4me1, and histone H3 K27 acetylation (H3K27ac). Of these, *MAP3K5*, *MET*, and *VEGFA* may be involved in inflammation-related energy metabolism *via* activation of the MAPK signaling cascades (55). miRNAs are short non-coding RNAs that can silence the expression of target genes by binding to the 3′-untranslated region of target mRNAs, leading to translational repression and/or mRNA degradation (56). They provide a rapid but reversible means of gene regulation, as a response to environmental stimuli at both the tissue and systemic levels without changing the DNA sequence itself (57).Over 2,500 mature human miRNAs have been identified, and they are thought to regulate up to 60% of human protein-coding genes (58). Some miRNAs are associated with chronic inflammation in T2DM; for example, miR-146a is downregulated in the serum of T2DM patients (59). Another miRNA associated with inflammation in T2DM is miR-147, which is overexpressed in the serum of diabetic and obese rats with periodontitis; miR-147 overexpression is believed to activate macrophages and increase the expression of pro-inflammatory markers such as TNF-α and IL-12 (60). In addition, chronic and transient hyperglycemia was also found to change the levels of miRNAs (miR-26a-5p, miR-26b-5p, let-7d-5p, let-7e-5p, miR-365a-3p, and miR-146a-5p) in adipocytes, which mostly converged to alter IL-6 transcription and can be instrumental in the development of inflammation and metabolic dysregulation of VAT (61).

## Epigenetic Remodeling of the Immune System in the Progression of T2DM

A growing body of literature links epigenetic modifications to crucial pathways in the pathogenesis of diabetes. As described above, besides altered metabolic processes, DNA methylation, covalent modification of histones, and the expression of non-coding RNA, in particular miRNAs, are also involved in the occurrence and development of chronic inflammation in diabetes. The epigenetic regulation of immune cell recruitment, proliferation, differentiation, and functional phenotypes plays a key role in promoting IR and impairing β-cell insulin production (**Table 1**).

**Table 1:** Inflammation-related Epigenetic modification Involved in T2DM.

Cell Type	Epigenetic Marks		Target	Position	Process(es)	Refs
Monocytes	DNA	hypomethylation	MCP-1 (CCL2)	blood	chemotaxis and recruitment	(62)
	histone	H3K9Ac	MCP-1 (CCL2)	blood	chemotaxis and recruitment	(63)
		H3K4me (3)	MCP-1 (CCL2)	blood	chemotaxis and recruitment	(63)
Macrophages	DNA	hypomethylation	Cfb, Serping1,Tnfsf15	muscle	activation of M1-Mϕs	(64)
		hypermethylation	Nrp1, Cxcr4, Plxnd1, Arg1, Cdk18, Fes	muscle	inhibtion of M2-Mϕs	(64)
		hypermethylation	PPAR-γ	vascular endothelium	activation of M1-Mϕs	(65)
	histone	H3K4me1	NF-κB		activation of M1-Mϕs	(66, 67)
		H3K9me3	IL-6		activation of M1-Mϕs	(68)
		HDAC3	STAT1		activation of M1-Mϕs	(69)
		HAT p300/CBP	HIF-1α	adipose	activation of M1-Mϕs	(70)
		HDAC7	TLR/NF-κB pathway	adipose	activation of M1-Mϕs	(71)
	microRNA	miR-10a-5p (↓)		adipose	activation of M1-Mϕs	(72)
		miR-34a (↑)	Klf4	adipose	inhibtion of M2-Mϕs	(73)
		miR-30 (↓)	Notch1 pathway	adipose	activation of M1-Mϕs	(74)
	LncRNA	Dnm3os (↑)	NKx3-2 AP1, STAT, IRF1		activation of M1-Mϕs	(75)
		HCG18 (↑)	TRAF6/NF-κB pathway	nerve	activation of M2-Mϕs	(76)
T cells	DNA	hypermethylation	CLSTN1	adipose	up-regulation in CD4+ cells	(77)
		hypermethylation	HGK		up-regulation of IL-6	(78)
	microRNA	miR-125b (↑)	Blimp-1, IRF-4		conversion of Tregs and Th2 cells into Th17 cells	(79)
		miR-326 (↑)		adipose	conversion of Th1 cells into Th17 cells	(80)
B cells	DNA	hypermethylation	LY86			(81)
	microRNA	miR-150 (↑)		adipose	activation of B cells	(82)

↑: up-regulated expression, ↓: down-regulated expression.

### Epigenetics Remodeling of Mϕs’ Recruitment and Functional Expression (Polarization) in T2DM

Although the pro-inflammatory M1 polarization of resident tissue macrophages is the trigger point causing chronic inflammation of the pancreas and diabetic peripheral glycometabolic tissue, chemotaxis and the recruitment of blood monocytes and macrophages mediated by CCL-2 are central factors in the subsequent inflammatory aggravation of metabolic tissues. A study investigating serum levels of CCL-2 in patients with DM and metabolic syndrome (MetS) found that serum CCL-2 levels were significantly increased in the MetS group and the DM group. Moreover, high CCL-2 levels showed a significant positive correlation with the typical clinical phenotypic features of DM such as high body mass index, waist-hip rate, triglyceride levels, and HOMA-IR (83). Then, through isolation of genomic DNA from peripheral blood mononuclear cells (PBMC) and methylation-specific polymerase chain reaction, the methylation status of CpG sites in the CCL-2 promoter was determined and the CCL-2 promoter was found to be hypermethylated in non-diabetic individuals (62). As the key players in the peripheral sensitization that leads neuropathic pain, CCL2 may be also associated with the diabetic peripheral neuropathy (DPN). Studies have revealed increased levels of H3K9Ac and H3K4me3 in the promoter regions of CCL2 genes in injured sciatic nerves, suggesting that CCL2 may be upregulated in injured peripheral nerves *via* epigenetic histone modification in infiltrating immune cells such as Mϕs (63).

After chemotaxis and recruitment into tissues, monocytes can differentiate into Mϕs gradually, and epigenetic remodeling are also participates in the upregulation of M1 polarization and inhibition of M2 polarization, together contributing to inflammation in T2DM. Varying patterns of methylation in Mϕ isolated from ischemic muscles were found between controls and hyperlipidemic T2DM patients. The promoters of *Cfb*, *Serping1*, and *Tnfsf15*, which are classically activated M1-Mϕs genes, were significantly hypomethylated, whereas the promoters of alternatively activated M2-Mϕs genes, including *Plxnd1*, *Arg1*, *Nrp1*, *Cxcr4*, *Fes*, and *Cdk18*, were significantly hypermethylated. Combined with the results of mRNA expression and immunohistochemistry, the predominance of proinflammatory M1-Mϕs over anti-inflammatory and proangiogenic M2-Mϕs was confirmed in hyperlipidemic and T2DM ischemic muscles (64). The polarization of Mϕs is also regulated by peroxisome proliferator-activated receptor γ (PPAR-γ), and DNMT1 can upregulate the DNA methylation status of the proximal PPAR-γpromoter and induce Mϕs to polarize into M1-Mϕs. Inhibition of DNA methylation in the PPAR-γpromoter by deleting 5-aza-2’-deoxycytidine or DNMT1 can promote selective activation of macrophages (m2-like macrophages), confirming the critical role of DNA methylation in PPAR-γ (65).

Besides DNMT1, DNMT3B is also an important regulator of Mϕs polarization; its expression is lower in alternatively activated M2-Mϕs, whereas a complete knockdown of DNMT3B is associated with a shift towards the M2 phenotype. Loss of function of DNMT3B is associated with decreased expression of inflammatory genes, including those encoding IL-1β and TNF-α, and impaired chemotactic ability (84).

Covalent modifications of histones, including methylation, acetylation, ubiquitination, phosphorylation and lactylation, have been shown to influence the function of Mϕs. Hyperglycemia can promote Mϕs activation *via* histone methylation of NF-κB. Advanced glycation end products (AGEs), the pathological product of hyperglycemia, can promote M1 activation of mouse primary Mϕs and increase the expression of nitric oxide synthase (NOS)2, TNF-α, and IL-6 based on the upregulation of the RAGE/NF-κB pathway (85). Histone methylation is involved in the regulation of M1-Mϕs mainly by modifying NF-κB expression. Thus, the transient hyperglycemia model revealed increased activation of H3K4 methylation mediated by the SETD7 (66) and SETD9 (67) in the promoter of the NF-κB p65 subunit. Li et al. found that SETD7/9 promotes the recruitment of p65 in human monocytes, thus regulating the expression of NF-κB target genes (86). SETD7/9 knockdown can inhibit TNF-α, IL-8 and CCL2 expression in human monocytes upon stimulation with TNFs. Hyperglycemia decreases H3K9me3 at the IL-6 promoter in human monocytes, thereby increasing IL-6 expression (68). In addition, the differentiation of monocytes to macrophages and the development of tolerance or trained immunity are closely related to the acquisition of distinct epigenetic signatures, such as H3K4me1, H3K4me3, and H3K27ac, in the promoter and enhancer regions (87). In addition, demethylation of H3K27 and H3K4me3, respectively, are involved in the M2 polarization and the expression of inflammatory cytokines produced by M1-Mϕs (88).

Histone acetylation was the first reported post-translational histone modification. The key enzymes, HATs and HDACs, which mediate the process of its modification, play an important role in the regulation of chemotaxis, recruitment, and polarization of Mϕs. HDAC 2, 3, 6, 7, and 9 can activate M1 polarization, and HDAC3, 4 can inhibit M2 polarization, leading to a pro-inflammatory status. Furthermore, SIRT2 upregulation can activate M2 polarization, while SIRT1, HDAC1, 4, 5, 7 can inhibit M1 polarization (89). Studies have shown that the class I HDACs are primarily involved in innate immunity and work by modulating genes regulated by Toll-like receptors (TLRs) and IFN (90). In this process, there is a bidirectional regulation characterized by the activation of the IFN pathway and inhibition of the TLR-NF-κB pathway. HDAC1 plays a vital role in STAT1- and/or STAT2-dependent IFN signaling. The recruitment of HDAC1 and the its interaction with STAT5 can, in turn, deacetylate CCAAT/enhancer-binding protein-β, thereby activating the transcription of interferon-stimulated genes (ISGs) and inducing the secretion of IFN (91). However, HDAC1 is a negative feedback regulator of the TLR-NF-κB pathway because it inhibits the promoter activity of TLR-induced genes, such as cyclooxygenase 2 (*Cox-2*), IL-12 subunit p40 (*IL-12p40*), and *IFN-β (* 90), and interacts directly with NF-κB p65 to inhibit its expression. HDAC3 also regulates inflammatory genes in Mϕs. Recent studies have shown that HDAC3 can regulate the transcription and expression of STAT1 by interacting with the transcription factor FOXK1 and forming a transcriptional complex that aggregates at the same position in the Stat1 promoter while simultaneously maintaining the stability of FOXK1, and activating the transcription of ISGs (69). In mice, M2 polarization was inhibited by HDAC3 through the repression of several Il4-related genes (92). In T2DM, HDAC3 has been suggested to promote M1 polarization and aggravate inflammation, and its activity is significantly and positively correlated with high levels of HbA1c and insulin, as well as that of circulating TNF-α and IL-6 (93). A hypoxic environment induced by the hyperproliferation of adipose tissue induces the M1 activation in T2DM patients. In addition,acetylation of hypoxia-inducible factor (HIF)-1α is mediated by the HAT p300/CBP (70), and upregulation of HDAC7 can also coordinate with HIF-1α to activate the TLR-NF-κB pathway (71).

Notably, because of hypoxic conditions, activation with pro-inflammatory stimuli such as LPS or IFN-γ, and increased ATP demand for active proliferation and differentiation, the metabolic reprogramming of macrophages is activated and switched from oxidative phosphorylation to glycolysis to generate ATP. This phenomenon is called the Warburg effect and causes the production and enrichment of large amounts of lactic acid in macrophages (94, 95). A recent study by Zhang et al. established a new function of lactate, which could contribute to a novel form of histone modification, histone lactylation, and the promotion of M2 polarization in Mϕs. Zhang et al. suggested that lactate can generate lactyl-CoA, which provides a lactyl group to the lysine tails of histone proteins through acetyltransferase p300, resulting in a modification called lactyllysine, leading to an M2-like phenotype (96). Recent studieshave identified that class I HDACs (HDAC1-3) are also the potential histone lysine delactylases (97). Although the exact changes in T2DM as a new histone modification have not been revealed, it is a potential explanation for the high lactate levels in diabetes and macrophage-mediated metabolic inflammation.

Non-coding RNAs, particularly miRNAs, play crucial roles in the regulation of the M1 and M2 polarization through targeting various adaptor proteins and transcription factors. During the process of M1 polarization, miR-9, miR-21, miR-125b, miR-127, and miR-155 are upregulated. Of these, miR-9can suppress the anti-inflammatory response by inhibiting PPAR-δ expression. In addition, miR-125b, miR-127, and miR-155 target *Bcl6*, *C/EBP*, *SOCS1*, and *IRF4* and promote the M1 phenotype *via* the JNK and P13K/Akt1. For the activation of M2 polarization, miR-21, miR-124, miR-132, and miR-125a-5p all play key roles in the regulation of *SIRPb1*, *STAT3*, *AChE*, and *KLF4*. miR-146a and let-7c can also inhibit activation of the NF-κB pathway through *IRAK1*, *TRAF6*, *PAK1*, and *C/EBP-δ (* 98). In T2DM, miRNAs also play an important regulatory role in the interaction of glycometabolic tissue and Mϕs. For example, miR-10a-5p is an important negative regulator of inflammation in ATMs and a high-fat diet can reduce miR-10a-5p levels in ATMs. Treatment of mice with the miR-10a-5p mimic inhibited pro-inflammatory responses and enhanced glucose tolerance (72). In addition, miR-34a secreted by adipose cells can suppress M2 polarization by repressing Krüppel-like factor 4 (Klf4) expression. This relationship was verified in obese mice and patients (73). miR-30, another adipocyte-derived exosomal miRNA, is also downregulated in M1-Mϕs and activates the Notch1 pathway (74). In contrast, some miRNAs derived from Mϕs, such as miR-210 and miR-155, can also induce IR. miR-210 derived from ATMs accelerates diabetic pathogenesis in mice by regulating glucose uptake and mitochondrial complex IV activity by targeting NDUFA4 expression. miR-210 can also assist miR-155 in influencing the expression of PPAR-γ and GLUT-4 in 3T3-L1 cells (99).

Apart from miRNAs, lncRNAs, such as lncRNA Dnm3os, also regulate Mϕs polarization in T2DM. A previous study showed that decreased nucleolin and overexpression of Dnm3os can enhance promoter H3K9ac, recruit histone acetyltransferase, and activate histone acetylation to upregulate the expression of inflammatory genes, such as *NKx3-2*, *AP1*, *STAT*, and *IRF1 (* 75). Further studies showed that lncRNA HCG18 participates in the pathology of DPN and can competitively bind miR-146a and upregulate TNF receptor associated with factor 6 (TRAF6)/NF-κB pathway to promote M1 polarization and the secretion of inflammatory factors (76).

As summarized above, in the context of T2DM, epigenetic modifications regulate the recruitment and polarization of macrophages and establish the inflammatory infiltrating status of glyco-metabolic organs and the pancreas. Cytokines derived from M1-like Mϕs play a crucial role in contributing to IR and impaired insulin production. The most studied cytokine TNF-α compromises tyrosine phosphorylation in the insulin-signaling cascade, mainly of the insulin receptor substrate (IRS) protein. These phosphorylation inhibitory effects are regulated by TNFα-induced kinases such as IκB kinase (IKK), c-Jun N-terminal kinase (JNK), and atypical protein kinase C (aPKC), thereby preventing insulin signaling (100). TNF-α also enhances ceramide synthesis and lipolysis in adipocytes and inhibits PPARγ expression. A normal level of PPAR-γ is necessary to maintain insulin sensitivity, and the effects of ceramides on inhibiting AKT phosphorylation and insulin action are well known. Interestingly, IL-6 is also involved in the regulation of insulin signaling by affecting the phosphorylation of IRS-1. In liver tissues, IL-6 exerts its function by binding to the IL-6 receptor a chain (IL-6Ra) and GP130 signaling chain complex of the liver cell membrane initiating Janus kinase (JAK)2/STAT-3-dependent transcriptional activation of target genes such as SOCS-3 (101).. SOCS-3 is not only a negative regulator of IL-6 signaling but also inhibits insulin signal transduction at the IRS protein level and causes hepatic insulin resistance (102, 103). In addition to inhibiting insulin signaling pathways in adipose and liver tissues, M1 polarization of islet-resident macrophages also leads to islet dedifferentiation and the reduction of glucose-stimulated insulin secretion (GSIS) function. This is closely related to the activation of IL-1β, another representative cytokine, and downstream JNK activation. JNK can inhibit GSIS by inhibiting the phosphorylation activation of the IRS-1-PI3K-AKT pathway in β cells and also reduces the phosphorylation level of FOX1, which is responsible for maintaining the differentiation and functional expression of pancreatic β cells. This dysfunction leads to the dedifferentiation of islet β-cells, further aggravating impaired islet secretion (25). In addition, IL-1β has also been shown to promote IR in adipose tissues and the liver based on the impairment of insulin signaling pathways.

### Epigenetic Remodeling of T and B Lymphocytes in T2DM

The role of immune cells in the development of T2DM is notlimited to macrophages. Both T and B lymphocytes also play a central role in the inflammatory process and the development of IR (16, 17, 28). Like macrophages, lymphocytes can be divided into two mutually limiting populations with primarily pro-inflammatory functions or primarily regulatory functions (104). CD4+T cells can further differentiate into different subtypes, such as pro-inflammatory Th1, Th17, and anti-inflammatory Th2 and Treg cells, under the stimulation of different transcription factors (TFs), such as IRF-4 and Foxp3 (105). Th1 and Th17 cells secrete IFN-γ, IL-6, IL-17, TNF-α, and other inflammatory factors to promote M1 polarization of Mϕs and enhance their proinflammatory functions (106). IL-2 secreted by Th1 cells can also promote the proliferation of CD8+ T cells, which can induce macrophage activation and migration to adipose tissues by secreting MCP-1, MCP-3, and RANTES (16). B cells are also critical regulators of inflammation in T2DM and act by promoting proinflammatory T cell function and secreting proinflammatory cytokines (107). Several clinical studies have confirmed that the adaptive immune milieu skews towardss a pro-inflammatory phenotype in individuals with prediabetes or T2DM (108), and epigenetics are thought to play a regulatory role in this phenotypic change.

In a previous study (109), global DNA hypermethylation in B cells associated with IR was detected in the peripheral blood of individuals with obesity and T2DM, and this altered pattern was gene- and cytokine-specific. Another study also found cell type-specific DNA methylation differences in CD4^+^ and CD8^+^ T cells in women with obesity. In this study, the amount of visceral adipose tissue (VAT) was strongly associated with the methylation level of CD4^+^ cells, including those of the four CG sites in the CLSTN1 promoter, which may regulate its expression (77). The overexpression of DNMT3a was thought to be a possible reason for global DNA hypermethylation in lymphocytes from T2DM individuals as well as a possible reason for IR development (110). Higher methylation of the lymphocyte antigen 86 (*LY86*) gene and subsequent decrease in the expression of its encoding protein MD-1, was found to be significantly correlated with obesity, IR, and inflammatory markers in two genome-wide DNA methylation panels (81). MD-1, together with RP105 (a TLR family protein), as a complex expressed on immune cells, including B cells, macrophages, and dendritic cells, may serve as a negative regulator of TLR4 signaling in LPS response (111). It has also been found that peripheral blood T cells of T2DM show increased methylation of the HGK promoter, which in turn regulates the decreased expression of *HGK* in T cells and causes the subsequent upregulation of IL-6 (78). Besides, high FOXP3 methylationand low FOXP3 expression levels were found in AT mononuclear cells from obese individuals (80). FOXP3 is a key transcription factor involved in the development and function of Treg cells. These findings suggest an association between early metabolic dysfunction and alterations in methylation. Further studies are warranted to determine the functional significance of such methylation changes.

miRNAs are also essential for IR and obesity-associated inflammation. Recent studies have shown that miRNAs are actively involved in T-cell recruitment and differentiation. The expression level of miR-125b has been shown to0 be elevated in PBMCs from patients with T2DM (112). miR-125b binds to the 3′-untranslated region of Blimp-1 and IRF-4 messenger RNAs and attenuates the expression of Blimp-1 and IRF-4 (79), which drives the conversion of Tregs and Th2 cells into Th17 cells (113), thereby promoting inflammation of adipose tissues by releasing IL-17. This indicates that the alteration of miR-125b in patients with T2DM may be involved in the pathogenesis of T2DM, as the newly defined inflammatory Th17 subset has emerged as a crucial player in IR and T2DM progression (108). A greater abundance of miR-326 in individuals with obesity was also found to participate directly in the polarization of Th1 cells towards Th17 cells, promoting the inflammatory state in obesity-induced adipose tissues (80). In addition to T-cells,B-cells miRNAs also play crucial roles in obesity. For instance, miR-150 regulates obesity-induced inflammation and IR by controlling the activation of B cells and their interactions with other immune cells (82).

**Figure 2:**
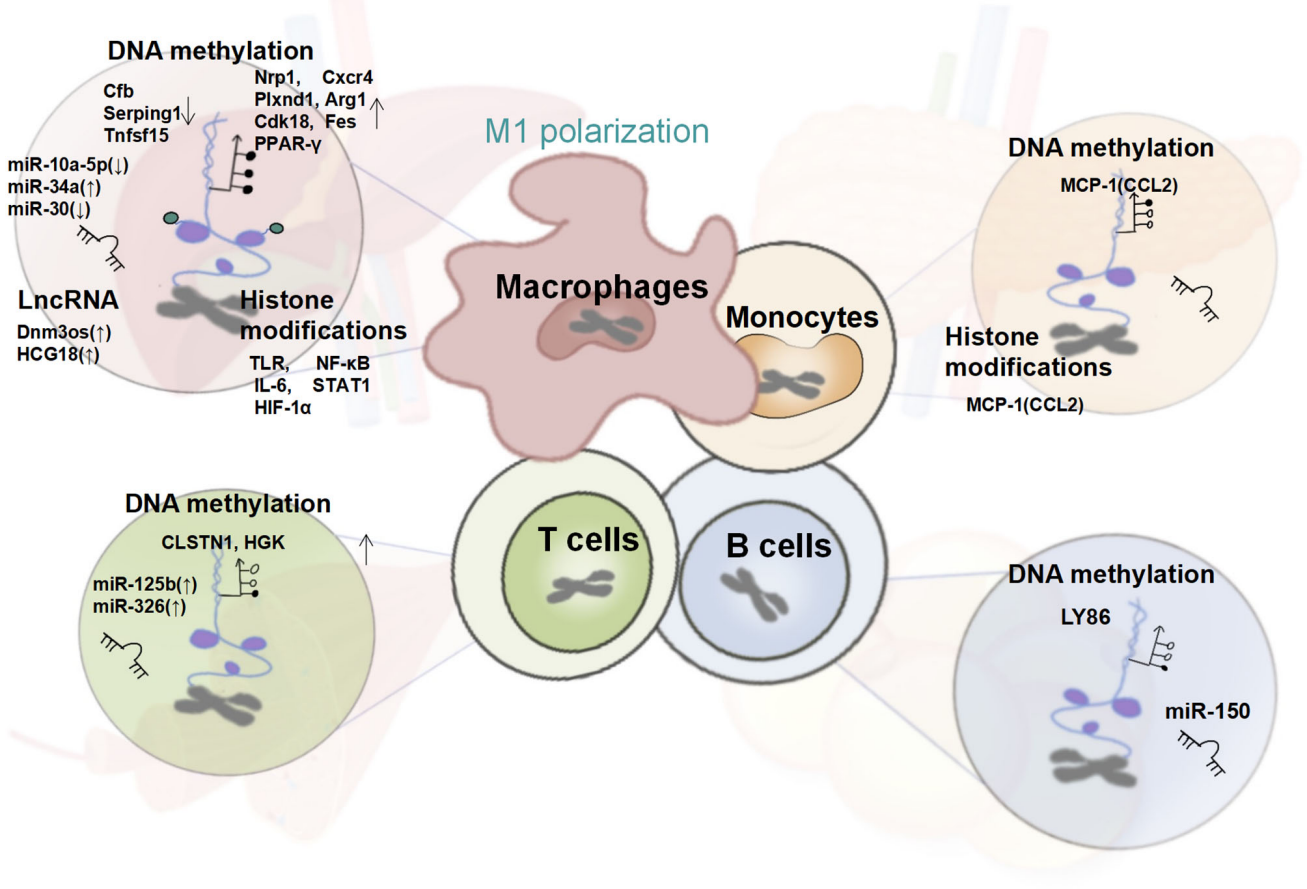
Changes in the Epigenetic Signature of Immune Cells in T2DM. In T2DM patients, the systemic and tissue-local inflammatory states mediated by the epigenetic regulation of monocytes and macrophages together with T cells, B cells plays a crucial role in insulin resistance. Altered global and gene-specific DNA methylation, histone modifications, as well as the expression of several non-coding RNAs are found to synergistically regulate the macrophage M1 pro-inflammatory phenotype, and the expression of genes encoding MCP-1/CCL-2, is up-regulated under the dual regulation of DNA methylation and histone modifications. Besides, the pro-inflammatory phenotype of T and B cells regulated by global and gene-specific DNA methylation and several miRNAs have also been reported in obese T2DM individuals.

Because of the proposed phenomenon of immunometabolism, increasing attention has been paid to the immune regulatory mechanism of T2DM. As described above, the cross-talk network centered on innate immune cells, such as the mononuclear-macrophage system, T cells, B cells, NK cells, and ILCs, can influence the function of islets, liver, skeletal muscle, and adipose tissue through its proliferation, recruitment, differentiation and functional phenotypes, such as the secretion of cytokines and chemokines, hence aggravating T2DM. In this process, epigenetic modifications play a key regulatory role. (**Figure 2**)

### Epigenetic Remodeling of ILCs May Also Participate in the Pathogenesis of T2DM

ILCs are a newly discovered class of lymphocytes that do not express diverse antigen receptors expressed on T and B cells. ILCs are almost tissue-resident cells, and their activation is mainly mediated by signals or cytokines expressed by tissue-resident cells and not antigenic stimulation. The function of ILCs is not only limited to classical immunology but also extends to metabolic homeostasis and tissue remodeling, among others. ILCs are mainly divided into three subtypes, namely, ILC1s, ILC2s, and ILC3s, which have similar functions to that of Th1, Th2, and Th17 cells (114). Therefore, ILCs play a key regulatory role in metabolic homeostasis, and an imbalance in their functions can induce T2DM. In adipose tissues, the homeostasis of both ILC1s and ILC2s is important for energy metabolism homeostasis. In the context of HFD-induced obesity, the production of IL-12 in adipose tissue leads to selective proliferation and accumulation of adipose-resident ILC1s based on the IL-12 receptor and STAT4. It can produce IFN-γ and establish a type 1 immune environment that drives proinflammatory M1 macrophage polarization to promote obesity-associated insulin resistance (115, 116). However, ILC2s showed the opposite effect. Under the stimulation of IL-25 and IL-33, they can produce IL-5 and IL-13, and establish an anti-inflammatory type 2 immune environment to drive downstream M2 macrophage polarization to alleviate insulin resistance. In addition, ILC2s could accelerate the beiging of adipocytes by upregulating the expression of methionine-enkephalin peptides and UCP-1, which could increase caloric consumption (117, 118). Furthermore, in addition to their key roles in VAT, ILC2s can induce dendritic cells to secrete retinoic acid, which promotes the secretion of insulin from β cells in the pancreas (119). Typical intestinal lamina propria ILC3s play an important role in resisting the imbalance of gut microbiota and maintaining intestinal mucosal homeostasis in obesity and T2DM, thus alleviating insulin resistance (120). Recent studies have shown that the proliferation of ILC3s and the upregulation of IL-22 secretion in the liver can alleviate liver inflammation and inhibit hepatocyte apoptosis caused by fat accumulation (121). It is worth noting that epigenetics remodeling, especially DNA methylation, also regulates the proliferation, differentiation, and functional phenotypes of ILCs. Previous research has revealed that different classes of ILCs are regulated by different TF such as T-BET (NK cells/ILC1s), EOMES (NK cells), GATA3 (ILC2s), and RORγt (ILC3s) (122). Based on whole-genome profiling of DNA methylation and hydroxymethylation, Peng et al. confirmed that there is differential promoter DNA methylation of key TFs. NK cell hypomethylation was primarily enriched for T-box TFs such as Tbx6, Tbx21, and Eomes, which are selectively expressed in NK cells. In ILC2s GATA motifs (Gata3 and Trps1) are primarily hypomethylated. Finally, hypomethylation of the ROR family (Rorc and Rora) has been observed in ILC3s (123). However, apart from DNA methylation, few studies have reported the regulatory functions of the covalent modification of histones and non-coding RNAs in ILCs and must be further studied. Considering the relatively recent discovery of its role in immune and metabolic homeostasis, ILC epigenetic modifications in the context of diabetes have not been revealed and require further exploration.

## Discussion and Perspectives

With the global pandemic of T2DM and the serious harm caused by its complications, studies to unravel the pathogenesis of diabetes and the development of new hypoglycemic drugs have continued. With the help of high-throughput sequencing methods such as genomics, transcriptomics, proteomics, and metabolomics, rapid screening of the key targets of diabetes has been achieved at multiple levels, from genes to downstream functional proteins and metabolites, and has enabled a more comprehensive and profound understanding of diabetes (124). Although the incidence of T2DM has increased dramatically over the past few decades (125), this short period is unlikely to cause significant changes in the human genome, possibly because the expression of key diabetes-related genes is regulated by environmental factors. Epigenetic modifications can reveal the effects of environmental factors such as diet, physical activity, stress, and temperature on T2DM. Since the first epigenetic study reported the changes in DNA methylation in pancreatic islets and skeletal muscle in T2DM individuals, numerous studies have revealed the many epigenetic modifications of islet mass and insulin secretion function, including in *PDX1*, *INS*, *ADCY5*, *CDKN1A*, *PDE7B*, *PPARGC1A*, among others. Besides, there are a number of epigenetic modifications that occur in target glycometabolic organs such as adipose tissue (in *ATP10A*, *IRS1*, *PPARG*, *JARID2*, *TCF7L2*, etc.), liver (in *ABCC3*, *GRB10*, *MOGAT1*, *PDGFA*, etc.), skeletal muscle (in *PPARGC1A*, *MAPK1*, *FADS2*, etc.), and blood (in *ABCG1*, *FAM123C*, *FHL2*, *KLF14*, etc.). Meanwhile, many studies have revealed the relationship between diet, physical activity, aging, and T2DM with respect to epigenetics (38).

It is worth noting that epigenetic information can also be inherited across several generations; this phenomenon is called intergenerational and transgenerational epigenetic inheritance (IEI and TEI). This indicates that environmental factors can influence not only individuals directly but also indirectly through their parents *via* IEI or TEI. Retrospective and prospective studies of human cohorts revealed that parental impact *via* overnutrition or undernutrition could deteriorate the metabolic health of their offspring (126). As shown in the Newborn Epigenetics Study (NEST) cohort, parental obesity can cause susceptibility to weight gain or obesity in offspring through altered small RNA and DNA methylation in human spermatozoa (127). In addition, DNA methylation of the offspring’s umbilical cord blood leukocytes has been identified (128). However, research on the relevant mechanisms of IEI or TEI is limited because of the requirement of long periods of human studies and ethical issues. The use of rodents as model systems enables overcoming some limitations of human studies. Increasing evidence has confirmed that paternal or maternal overnutrition, overweight, and T2DM could all increase offspring’s risk for obesity and diabetes. The core mechanisms by which IEI or TEI manifests its effects include the programming regulation of the offspring’s pancreas and adipose tissue by epigenetic inactivation of DNA methylation and microRNA in germ cells during the process of development and functional differentiation (126, 129). However, there are only a few studies on the mechanisms of IEI and TEI from an immunological perspective and should be studied to reveal the mechanisms of inherited metabolic disorders to explore prevention and treatment methods.

The inflammatory state caused by immune imbalance plays an important role in the development of T2DM. Therefore, epigenetic modifications of immune cells can indirectly regulate the inflammatory state of the body and affect insulin resistance and insulin secretion dysfunction.In our review, we focused on summarizing the changes in immune-related epigenetic modifications in the context of T2DM, especially the epigenetic regulation of macrophages, T and B lymphocyte recruitment, proliferation, differentiation, and functional phenotypes. In addition, we also identified some possible directions for future research, such as histone lactylation of macrophages in the background of T2DM and epigenetic modification of ILCs, which are widely involved in glucolipid metabolism and immune homeostasis. These findings provide novel insights for revealing the pathogenesis of T2DM from the perspective of epigenetic immune modification and can provide new directions to reveal the pathogenesis of T2DM with respect to epigenetics. However, several issues need to be addressed. For example, as it is difficult to obtain diabetic tissue samples, there are relatively few epigenetic studies based on the local tissues of patients, and most studies are carried out on the epigenetics of blood. Thus, larger cohorts must be conducted through worldwide collaborations, and owing to the tissue- and cell-specific nature of epigenetic modifications, more studies on human biopsies are desirable. Meanwhile, most of the studies at this stage are descriptive studies, and although they provide a macroscopic understanding of glycolipid metabolic functions and immune-related epigenetic changes in T2DM, the lack of detailed studies leaves us with uncertainty about its targeting, which further affects the application of research findings in clinical diagnosis and treatment.

The application of epigenetic findings facilitates the clinical diagnosis and treatment of T2DM. First, the prevention of T2DM, is crucial for the diagnosis and treatment of diabetes; therefore, the identification of epigenetic biomarkers indicative of T2DM susceptibility is of great significance. Notably, biomarkers need to be identified in easily accessible human samples, and blood samples are a good choice. Although blood epigenetic biomarkers have been used for tumor prediction, in T2DM, definitive epigenetic biomarkers still need to undergo extensive validation (130). Clinical translational research based on epigenetic reversibility has also been conducted, and some drugs that affect DNA methylation and histone modification, such as histone deacetylase inhibitors (valproic acid, sodium phenylbutyrate, vorinostat, and givinostat), histone acetyltransferase inhibitors (curcumin), protein arginine methyltransferase inhibitors (AMI-1), DNA methyltransferase inhibitors (hydralazine, procainamide, RG108, MG98), histone demethylating inhibitors (tranylcypromine), and sirtuin-activating compounds (resveratrol), have been developed (131). The efficacy and application of these drugs in alleviating metabolic diseases included obesity, diabetes, fatty liver, and metabolic syndrome have been preliminarily confirmed, and corresponding clinical studies, including those involving valproic acid, sodium phenylbutyrate, resveratrol, etc., are being carried out gradually. However, the low specificity and global action of these medicines may lead to side effects. Therefore, the development of highly specific epigenetic drugs targeting specific diseases and cell functions may be a more suitable direction for future research.

## Author Contributions

QD and ZG are co-responsible for the collection, collation, and writing of the original manuscript. LZ designed and revised the manuscript. KC, QZ, and SH are responsible for the concept development, revision, and review of the manuscript. All authors contributed to the article and approved the submitted version.

## Funding

This work was supported by the National Natural Science Foundation of China (Grant Nos. 81704067) and Innovation Team and Talents Cultivation Program of National Administration of Traditional Chinese Medicine (Nos.ZYYCXTD-D-202001).

## Conflict of Interest

The authors declare that the research was conducted in the absence of any commercial or financial relationships that could be construed as a potential conflict of interest.

## Publisher’s Note

All claims expressed in this article are solely those of the authors and do not necessarily represent those of their affiliated organizations, or those of the publisher, the editors and the reviewers. Any product that may be evaluated in this article, or claim that may be made by its manufacturer, is not guaranteed or endorsed by the publisher.
